# Imatinib-Associated Interstitial Lung Disease with a Fibrotic NSIP Pattern on Transbronchial Lung Cryobiopsy: A Case Report and Review of the Literature

**DOI:** 10.3390/reports9030303

**Published:** 2026-09-10

**Authors:** Pier-Valerio Mari, Lorenzo Carriera, Filippo Lococo, Stefano Margaritora, Simone Ielo, Veronica Ojetti, Giulio Onelli, Fabrizio Liberati, Vittorio Pietrangeli

**Affiliations:** 1 Internal Medicine, San Carlo di Nancy Hospital, 00165 Rome, Italy; 2 Department of Pulmonology and Sub-Intensive Respiratory Unit, Santa Maria della Misericordia Hospital, 06156 Perugia, Italy; 3 Faculty of Medicine and Surgery, Università Cattolica del Sacro Cuore, 00168 Rome, Italy; 4 Department of General Thoracic Surgery, Fondazione Policlinico Universitario “A. Gemelli” IRCCS, Università Cattolica del Sacro Cuore, 00168 Rome, Italy; 5 UOC Pneumologia e UTIP, Ospedale San Donato, USL Toscana SudEst, 52100 Arezzo, Italy; 6 UniCamillus International Medical University of Rome, 00131 Rome, Italy; 7 Interventional Pulmonology Unit, Department of Pulmonology, San Camillo de Lellis Hospital, ASL Rieti, 02100 Rieti, Italy; 8 Unit of Pathological Anatomy, San Camillo de Lellis Hospital, ASL Rieti, 02100 Rieti, Italy

**Keywords:** chronic myeloid leukemia, cryobiopsy, drug-induced interstitial lung disease, imatinib, non-specific interstitial pneumonia, tyrosine kinase inhibitor

## Abstract

**Background and Clinical Significance**: Interstitial lung disease (ILD) is an uncommon but potentially serious complication of imatinib. Its histopathological characterization has so far relied on surgical biopsy or transbronchial forceps sampling, and no case characterized by transbronchial lung cryobiopsy (TBLC) has been reported. The single previous description of a non-specific interstitial pneumonia (NSIP) pattern with imatinib came from a surgical specimen and was cellular in type, with complete radiological resolution after withdrawal. **Case Presentation**: A 69-year-old never-smoker began imatinib 400 mg daily for Philadelphia chromosome-positive chronic myeloid leukemia; chest computed tomography performed immediately beforehand was normal. Approximately three months later, she developed dyspnea, dry cough and a cutaneous rash, progressing to acute respiratory failure with forced vital capacity 49% and diffusing capacity 30% of predicted. Avian exposure had ceased one month before symptom onset, and she had never received amiodarone. Although serum precipitins were unavailable, hypersensitivity pneumonitis was considered less likely given the temporal relationship but could not be definitively excluded. TBLC yielded specimens showing fibrotic thickening of the alveolar septa by hyaline collagen deposition with a focal lymphomonocytic infiltrate, corresponding to a fibrotic NSIP pattern. After drug withdrawal and administration of corticosteroids, forced vital capacity rose to 82% of predicted. Multidisciplinary discussion concluded that this was drug-induced ILD, graded probable on the Naranjo scale. Imatinib was subsequently replaced by bosutinib for insufficient molecular response, without pulmonary recurrence. **Conclusions**: Imatinib-associated ILD may present with an organizing-pneumonia-like radiological pattern followed by a fibrosing pattern, with cryobiopsy performed later demonstrating fibrotic NSIP. Because no tissue was obtained during the acute phase, this sequence remains a hypothesis rather than a demonstrated evolution. Cryobiopsy can be performed safely when surgical biopsy is not appropriate.

## 1. Introduction and Clinical Significance

Drug-induced interstitial lung disease (DI-ILD) has an estimated incidence of between 4.1 and 12.4 cases per million per year and accounts for approximately 3% to 5% of diagnoses in interstitial lung disease registries and cohorts [1,2]. Its profile has changed with the expansion of targeted anticancer therapy [1]. Close to 400 compounds have now been implicated, and the number continues to rise [2]. Diagnosis relies on temporal association with exposure, a compatible clinicoradiological and histological pattern, meticulous exclusion of alternatives and improvement after withdrawal [3]. Imatinib transformed the prognosis of chronic myeloid leukemia (CML), and its long-term tolerability is generally excellent. Pulmonary toxicity is nonetheless recognized: among Bcr-Abl tyrosine kinase inhibitors (TKIs), pleural effusion is most often linked to dasatinib and bosutinib and pulmonary arterial hypertension is linked to dasatinib, whereas ILD has been reported predominantly with imatinib [4]. Sixteen of twenty-eight clinically used tyrosine kinase inhibitors have been implicated in ILD [5]. The largest early series described 27 cases [6], and individual reports have accumulated since [7,8,9,10,11]. Reported incidence is approximately 0.5% in the largest series [6,9]. What remains scarce is tissue. Because DI-ILD is a diagnosis of exclusion with no pathognomonic feature, histology contributes chiefly by excluding alternatives and by placing the injury within a recognized morphological pattern. Transbronchial lung cryobiopsy (TBLC) yields specimens large enough for architectural assessment at a lower risk than surgical biopsy, and its diagnostic value increases when interpreted within multidisciplinary discussion [12]. A small series of DI-ILD cases characterized in this way has begun to appear (bispecific T-cell engagers [13], abemaciclib [14], ixekizumab [15], minodronic acid [16]), but to our knowledge imatinib has not been characterized in this way. Histological information on imatinib-associated ILD is limited to a handful of specimens. In the largest series, transbronchial forceps biopsy in five patients and video-assisted thoracoscopic surgery in one showed interstitial inflammation and fibrosis, intraluminal organizing fibrosis and lymphoplasmacytic alveolitis, without the descriptor NSIP being applied [6]. A single surgical biopsy has since been reported as compatible with NSIP in its cellular form and with complete radiological resolution after withdrawal [9]. We report a case of imatinib-associated ILD characterized by TBLC, which is, to our knowledge, the first to present a fibrotic rather than cellular NSIP pattern, and use it to examine how causality is established when infection, drugs and empirical treatment converge within the same window. For the accompanying literature review, PubMed was searched from inception to August 2026 using combinations of “imatinib” with “interstitial lung disease,” “pneumonitis,” “organizing pneumonia,” and “pulmonary toxicity,” without language restrictions. The reference lists of retrieved articles were also screened manually to identify additional relevant reports. Publications providing individual-level clinical details were included and are summarized in a dedicated table.

## 2. Case Presentation

A 69-year-old woman, a lifelong never-smoker with no occupational exposure to dust or solvents and no prior respiratory disease, was diagnosed with Philadelphia chromosome-positive CML in July 2025 (BCR-ABL major BCR transcript positive, minor BCR negative). A chest computed tomography (CT) performed on 28 July 2025, before any antineoplastic treatment, was normal, with no interstitial or airspace abnormality. Imatinib at 400 mg once daily was started in August 2025. Her medical history included atrial fibrillation treated with ablation, moderate mitral regurgitation following minor chordal rupture, arterial hypertension, dyslipidemia, total thyroidectomy for toxic multinodular goiter, gastro-esophageal reflux disease with biopsy-proven Barrett’s esophagus, and hiatal hernia. Concomitant medications were apixaban, losartan, metoprolol, furosemide, pantoprazole and folic acid. She had never received amiodarone or any other antiarrhythmic drug associated with pulmonary toxicity. She lived in a rural area and kept poultry; this exposure had ceased in October 2025. In November 2025, she experienced a self-limiting influenza-like illness, after which progressive exertional dyspnea developed. High-resolution CT on 30 December 2025 showed extensive peribronchial consolidative opacities in both upper lobes and in the pyramidal segments of the lower lobes, without effusion, raising the possibility of alternatives such as sarcoidosis or organizing pneumonia. At a pulmonological assessment on 7 January 2026, she reported dyspnea, dry cough, low-grade fever and a cutaneous rash that had appeared during imatinib therapy. Oxygen saturation was 91% on room air. Pulmonary function was severely impaired: forced vital capacity (FVC) was 49% predicted, forced expiratory volume in one second (FEV_1_) was 46%, FEV_1_/FVC was 0.74, total lung capacity (TLC) was 55%, single-breath diffusing capacity for carbon monoxide (DLCO) was 30% and DLCO/alveolar volume (DLCO/VA) was 45% (Table 1).

**Figure 1:**
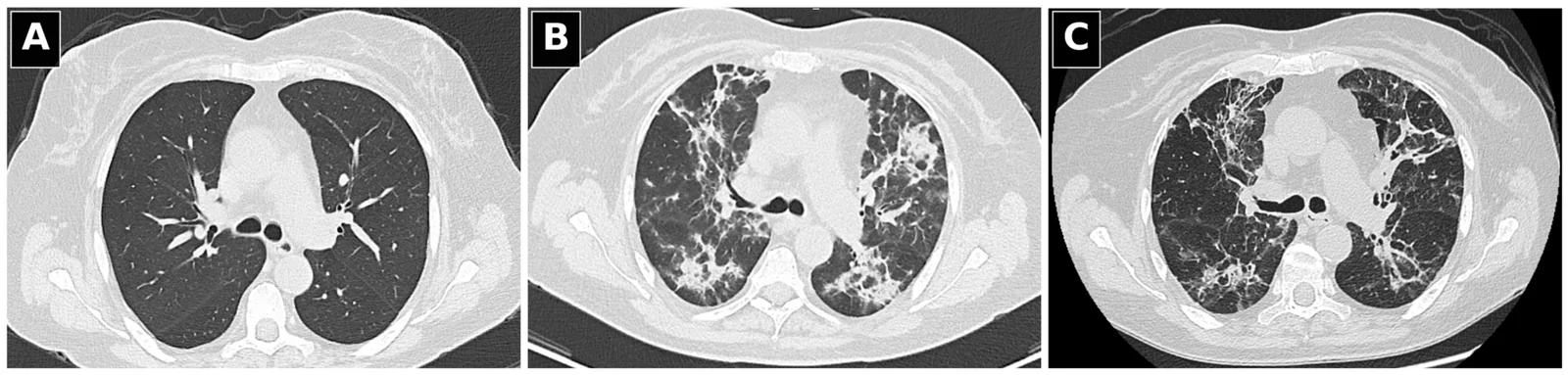
Serial axial chest computed tomography at the level of the tracheal bifurcation, lung window. (**A**) On 28 July 2025, before starting imatinib: normal lung parenchyma, with no interstitial or airspace abnormality. (**B**) On 30 December 2025, approximately five months after starting imatinib and one month after symptom onset: extensive bilateral peribronchovascular consolidative opacities with irregular margins, predominantly in the upper lobes, without pleural effusion, in an organizing-pneumonia-like distribution. (**C**) On 11 February 2026, at the time of acute respiratory failure: coarse reticulation with interlobular and intralobular septal thickening, traction bronchiectasis and architectural distortion superimposed on ground-glass attenuation, indicating evolution towards a fibrosing pattern.

QuantiFERON testing was negative. On 11 February 2026, she presented to the emergency department with worsening dyspnea. CT showed smooth reticular thickening of the interlobular and intralobular septa involving nearly all lobes with upper-lobe predominance, apical traction bronchiectasis, tree-in-bud opacities and ground-glass attenuation with a crazy-paving appearance, interpreted as possible fibrosing ILD (Figure 1).

**Figure 2:**
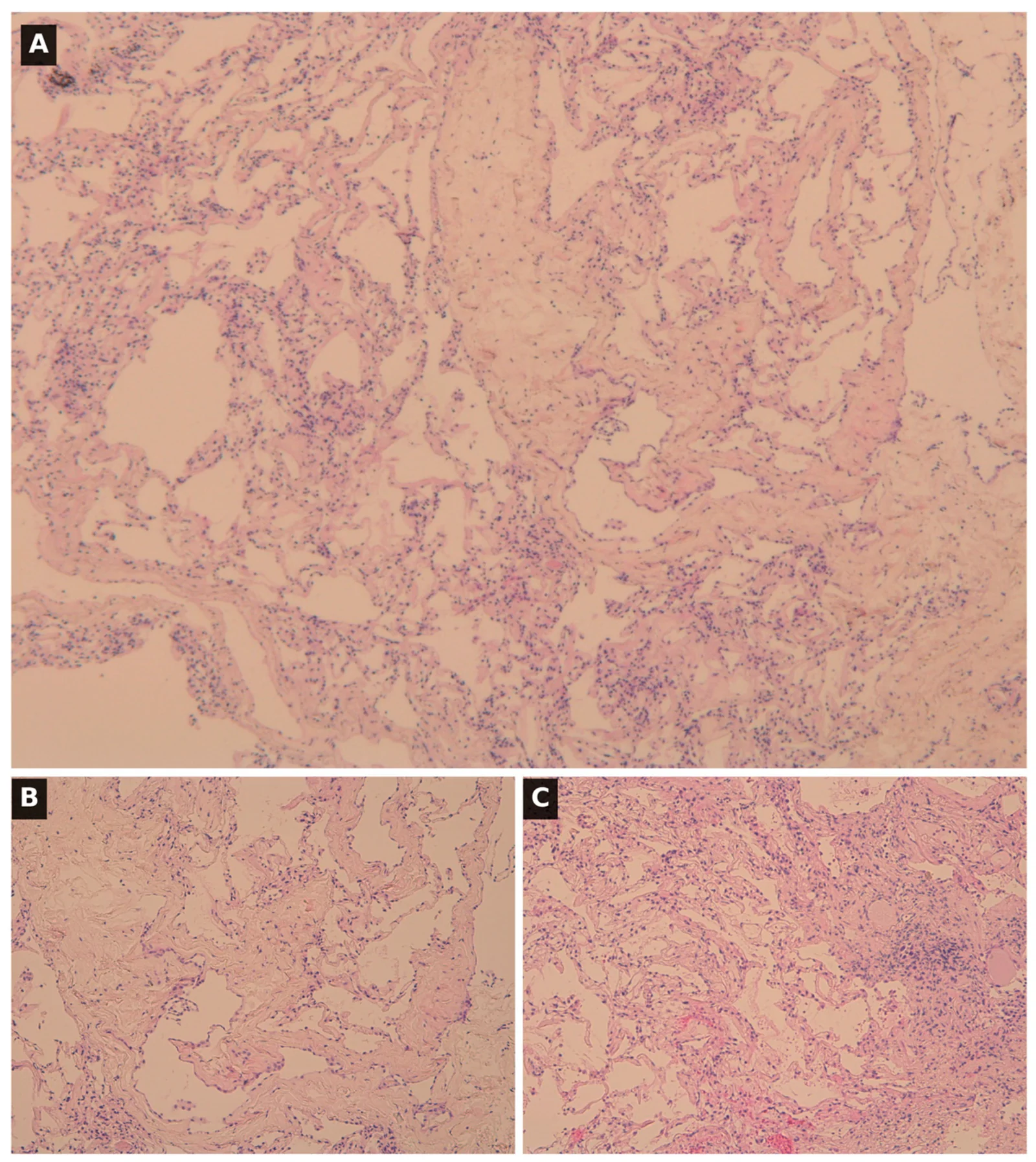
Transbronchial lung cryobiopsy specimens from the ventral branch of the left upper lobe (hematoxylin and eosin). (**A**) Low-power view (4×) showing diffuse fibrotic thickening of the alveolar septa with preserved overall architecture. (**B**) Higher magnification (10×) of a fibrotic area demonstrating septal widening by hyaline collagen deposition. (**C**) Fibrotic septal thickening with a focal lymphomonocytic inflammatory infiltrate (10×). No granulomas, multinucleated giant cells, fibroblastic foci, hyaline membranes or intraluminal organizing plugs were identified.

She was admitted from 15 February to 4 March 2026. Inflammatory markers were unremarkable (C-reactive protein 0.5 mg/dL). Arterial blood gas on room air showed pH 7.44, PaCO_2_ 44.2 mmHg, and PaO_2_ 75.3 mmHg. Echocardiography demonstrated preserved left ventricular systolic function, grade I diastolic dysfunction, mild-to-moderate mitral regurgitation and no pericardial effusion, excluding a cardiogenic contribution. Sputum culture and nasopharyngeal swabs for SARS-CoV-2, respiratory syncytial virus and influenza A and B were negative. Flexible bronchoscopy with bronchoalveolar lavage (BAL) was performed; multiplex PCR detected rhinovirus/enterovirus, metapneumovirus and parainfluenza virus, and culture grew filamentous fungi, while stains for acid-fast bacilli were negative. Cryobiopsy was deferred because of the perceived procedural risk. She received piperacillin–tazobactam, systemic corticosteroids and supplemental oxygen, with progressive clinical improvement and weaning from oxygen. The referring hematology center advised interruption of imatinib, which was withheld from 2 March 2026. The discharge diagnosis was transient acute respiratory failure attributed to probable viral infection occurring in suspected ILD versus drug-induced pneumopathy, pending further investigation. Prednisone 37.5 mg daily was prescribed from 4 March 2026. CT on 12 March 2026 showed moderate regression of the peribronchial thickening and of the tree-in-bud opacities, with stable parenchymal distortion and apical fibrotic change. Imatinib was resumed on 1 April 2026. This was not an intentional diagnostic rechallenge: the drug was restarted to maintain treatment of the underlying leukemia while the pulmonary diagnosis was still being clarified. Pulmonary function on 7 April 2026 had improved markedly: FVC was 82% predicted, FEV_1_ was 79%, TLC was 75%, with DLCO/VA unchanged at 44% (Table 1). Multiple-breath nitrogen washout showed a markedly elevated lung clearance index of 10.53. Although the patient had improved both clinically and functionally, histological characterization was still considered necessary. The referring hematology center required a definitive pulmonary diagnosis before deciding whether tyrosine kinase inhibitor treatment could be continued or changed, and the radiological abnormalities had not resolved, leaving the distinction between drug-induced disease and independent fibrosing interstitial pneumonia unsettled. Cryobiopsy, deferred during the acute episode because of the perceived procedural risk, was therefore performed once the patient was stable enough to tolerate rigid bronchoscopy under general anesthesia. On 27–28 April 2026, the patient underwent rigid bronchoscopy under general anesthesia. Apixaban had been withheld for 48 h. BAL from the ventral branch of the right upper lobe yielded 80% macrophages, 16% lymphocytes, 3% neutrophils and less than 1% eosinophils, with no malignant cells. Two transbronchial cryobiopsies were obtained from the ventral branch of the left upper lobe, with a Fogarty balloon positioned prophylactically and adrenaline instillation for hemostasis. No periprocedural complication occurred, and chest radiography excluded pneumothorax. Published cryobiopsy protocols advise withholding direct oral anticoagulants for approximately seven days [17]; the shorter interval used here was agreed after multidisciplinary assessment of thrombotic risk. The specimens measured 0.4 and 0.3 cm and showed diffuse fibrotic thickening of the interalveolar septa by deposition of hyaline collagen, with a minimal and focal lymphomonocytic infiltrate; one fragment was partially lined by pleura. The appearances corresponded to a fibrotic non-specific interstitial pneumonia (NSIP) pattern (Figure 2).

The case was reviewed by the interventional pulmonology multidisciplinary group, which concluded that the findings represented drug-induced ILD with a fibrotic NSIP pattern. Prednisone was replaced by deflazacort at 30 mg daily. The dose was halved to 15 mg daily on 4 June 2026, and on 2 July 2026 a further reduction to 7.5 mg daily for three months before withdrawal was planned, giving a projected total corticosteroid exposure of approximately eight months from March 2026. On 21 May 2026, imatinib was discontinued by the treating hematologist because of insufficient molecular response, and bosutinib 300 mg daily was commenced the following day. Molecular assessment at diagnosis had shown a major BCR-ABL transcript, corresponding to p210, with minor BCR negative. Subsequent quantitative monitoring of BCR-ABL transcript levels was performed at the referring hematology center and the values were not available to the authors; the judgement that the molecular response was insufficient and the consequent decision to switch were therefore made by the treating hematologist on data we could not independently verify. Kinase domain sequencing was not performed. Pulmonary function in June 2026 showed FVC at 73% predicted, FEV_1_ at 68%, DLCO at 67% and DLCO/VA at 56%; the six-minute walk distance was 330 m with a resting saturation of 95%, a mean of 89.3% and a nadir of 83%. Ambulatory oxygen was prescribed. Corticosteroid tapering to withdrawal was planned, with CT and pulmonary function reassessment at six months. At the most recent review, the patient was asymptomatic and free of dyspnea after approximately two and a half months of bosutinib, with no pulmonary recurrence.

## 3. Discussion

This case adds imatinib to the small group of drugs whose pulmonary toxicity has been characterized by cryobiopsy, and it illustrates the more general problem of attributing an interstitial process to a drug when several plausible causes occupy the same interval. The main clinical, radiological, histopathological, and outcome characteristics of previously reported cases of imatinib-associated ILD are summarized in Table 2.

**Table 2:** Reported cases of imatinib-associated interstitial lung disease.

First Author, Year	*n*	Age, Sex	Indication	Imatinib Dose (mg/Day)	Latency	Radiological Pattern	Biopsy Method	Histopathology	Dechallenge	Rechallenge	Outcome
Ohnishi 2006 [6]	27 (aggregate)	median 63 (47–82); 17 M/10 F	CML 23, GIST 4	median 400 (200–600)	median 49 d (10–282)	HR 30%, IP 26%, COP 15%, PBVB 15%, nodular 11%; no DAD	TBLB 5, VATS 1	Interstitial inflammation and fibrosis; intraluminal organizing fibrosis; alveolitis with lymphocytes and plasma cells; type II pneumocyte hyperplasia. NSIP not used as a descriptor	Withdrawn in all; 24/27 corticosteroids	Re-administered at reduced dose in 11; recurrence in 4 (36%)	Complete resolution 7, improvement 16, no improvement 4, fibrosis 1
Isshiki 2004 [7]	1	78 F	CML chronic phase	400	~1 month	Bilateral reticular opacities along bronchovascular bundles extending subpleurally; interlobular septal thickening; bilateral pleural effusions	Not performed	NR	Withdrawn; prednisolone	Not performed	Improved but not fully resolved at ~11 months
Carrillo-Esper 2009 [8]	1	48 F	CML, blast crisis	400 for 4 years, then 800	2 months after dose escalation to 800 mg	Bilateral consolidation with air bronchogram; right-predominant pleural effusion	BAL only (scant monocytes; no organisms or malignant cells)	NR	Withdrawn; methylprednisolone 250 mg/day; mechanical ventilation	Not performed	Marked improvement at 72 h; extubated; radiological resolution
Lee 2015 [9]	1	41 F	Recurrent rectal GIST	400	8 months	Multiple ground-glass opacities and irregular nodules, lower-lobe predominant	VATS wedge resection	Chronic interstitial inflammation with lymphoid hyperplasia and histiocyte aggregation; compatible with NSIP (CELLULAR)	Withdrawn; no corticosteroid reported	Not performed	Cough resolved within weeks; complete radiological resolution at 1 month
Zhang 2019 [11]	1	49 M	CML chronic phase	400	9 months	Dense cord- and grid-shaped opacities along bronchi, upper-lobe predominant	Not performed	NR	Withdrawn; prednisone 0.25–0.5 mg/kg/day	YES—dyspnoea and cough recurred within 2 weeks of reintroduction	Switched to nilotinib; lung function improved (DLCO 51.9 → 59.3%) but irreversible fibrosis on CT at 29 months
Tamura 2013 [18]	1 (of 66 DIPT)	NR	NR	NR	NR	HP-like pattern with centrilobular nodules	TBLB or VATS	Granuloma or organizing tissue in random distribution, independent of the respiratory bronchiole	NR	NR	NR
Guzelkucuk 2017 [10]	1	16 M	CML chronic phase	NR	20 days	Nodules, air bronchograms, ground-glass in lateral and posterior basal segments; small pleural effusions	Not performed	NR	Withdrawn; IV methylprednisolone 2 mg/kg	Not performed	Fever controlled within 4 h; radiological normalization at 5 days; dasatinib thereafter without recurrence
PRESENT CASE	1	69 F	CML chronic phase, Ph+	400	~3 months (August 2025 → November 2025)	OP-like pattern at onset (peribronchial consolidation) evolving to IP pattern (reticulation, traction bronchiectasis, crazy paving)	TRANSBRONCHIAL LUNG CRYOBIOPSY	Fibrotic thickening of alveolar septa with hyaline collagen deposition and focal lymphomonocytic infiltrate; FIBROTIC NSIP	Withdrawn 30 days; deflazacort	Re-exposed after 50 days at full dose—no unequivocal recurrence	FVC 49% → 82% predicted; residual fibrosis; bosutinib tolerated

NR, not reported; GIST, gastrointestinal stromal tumor; TBLB, transbronchial lung biopsy; VATS, video-assisted thoracoscopic surgery; HR, hypersensitivity reaction; IP, interstitial pneumonia; COP, cryptogenic organizing pneumonia; OP, organizing pneumonia; PBVB, peribronchovascular bundle; DAD, diffuse alveolar damage.

### 3.1. Why the Drug, and Not Something Else

Three features carry most of the weight. First, a chest CT obtained days before the first dose was normal, which places the onset unambiguously after exposure and makes a pre-existing subclinical ILD untenable. Second, avian exposure ceased in October 2025, one month before the first symptoms and two months before the first abnormal CT, and the disease appeared and progressed after that exposure had already ended. This chronology makes hypersensitivity pneumonitis less likely, but we do not regard it as excluded. Clinical and radiological manifestations may become apparent, persist or progress after antigen removal, particularly in subacute and fibrotic forms and after substantial prior exposure, and serum precipitins against avian antigens were not obtained. The histological findings are compatible with fibrotic hypersensitivity pneumonitis, although no granulomas, multinucleated giant cells or peribronchiolar cellular bronchiolitis were identified, and an HP-like radiological pattern was itself described with imatinib [18]. Hypersensitivity pneumonitis therefore remains a differential diagnosis that could not be formally excluded, and this is a limitation of the present report. Third, the patient never received amiodarone or any other antiarrhythmic with recognized pulmonary toxicity, and echocardiography excluded a cardiogenic contribution. A cutaneous rash concurrent with the pulmonary presentation adds an extrapulmonary hypersensitivity manifestation consistent with drug etiology.

### 3.2. Rechallenge in the Published Experience

Imatinib was withheld for thirty days and then resumed at full dose for fifty days without unequivocal recurrence. This might be read as evidence against attribution, but the largest published series argues otherwise: of the eleven patients re-administered imatinib at a reduced dose after recovery, ILD recurred in only four [6]. Absence of recurrence on re-exposure is therefore the dominant behavior rather than an exculpating finding, and a positive rechallenge such as that reported by Zhang and colleagues [11] is the exception. We assigned a score of zero to the Naranjo rechallenge item because the absence of recurrence after re-exposure does not warrant a positive score. However, a negative rechallenge still weakens the causal association, although it does not exclude it, and is one reason why causality in this case was considered probable rather than definite.

### 3.3. The Alternatives Examined

Three findings might be advanced against drug etiology, and none survives scrutiny. Multiplex PCR on bronchoalveolar lavage detected rhinovirus/enterovirus, metapneumovirus and parainfluenza virus, and a self-limiting influenza-like illness preceded symptom onset by weeks. These findings deserve careful weighting. Multiplex PCR detects viral nucleic acid rather than replicating virus, and prolonged shedding after a resolved upper respiratory infection is well recognized, particularly for rhinovirus; the simultaneous detection of three agents in a patient whose nasopharyngeal testing at presentation was negative and whose inflammatory markers remained normal argues against concurrent active viral pneumonia. We nevertheless consider it likely that a viral infection contributed to the acute deterioration of February 2026, superimposed on an interstitial process that was already established radiologically two months earlier and that had begun while the drug was being taken. What the viral findings do not explain is the course: the respiratory failure was transient, whereas the interstitial abnormality persisted for months and left traction bronchiectasis and established septal fibrosis. Filamentous fungi grew from a single culture. No species identification or serum galactomannan was obtained, the radiological appearances showed none of the features of invasive fungal disease, the patient was not neutropenic, and no antifungal therapy was given; the growth was therefore interpreted as airway colonization or contamination rather than infection, an interpretation supported by the subsequent clinical and functional recovery without antifungal treatment. Finally, corticosteroids were started two days after imatinib was withheld, so the two interventions cannot be disentangled from one another, but neither can either be dismissed, and functional recovery was subsequently maintained through corticosteroids, tapering to withdrawal.

### 3.4. The Rechallenge and the Naranjo Score

During the 50-day period of re-administration, FVC decreased from 82% to 73% predicted while corticosteroids were being tapered; over the same interval, however, DLCO/VA increased from 44% to 56%. The decline in FVC should therefore be interpreted cautiously. The two measurements were obtained in different laboratories, using different reference equations and different techniques for lung-volume assessment, and a difference of this magnitude may fall within the expected range of methodological and interlaboratory variability. We therefore do not consider this change to provide convincing evidence of a biologically meaningful deterioration. Genuine recurrent alveolar injury would not be expected to improve gas transfer, and we therefore did not score this as a positive rechallenge. Causality is accordingly graded probable rather than certain, with a Naranjo score of seven, applying the established framework for drug-induced respiratory disease [3]. Probable is the standard verdict in drug-induced ILD, where a definite rating requires a positive rechallenge that is neither ethical nor often available; it reflects the evidentiary structure of the diagnosis rather than a weakness of this case.

### 3.5. Dissociation Between Lung Volumes and Gas Transfer

Between January and April, lung volumes recovered substantially while DLCO/VA remained essentially unchanged; the improvement in single-breath DLCO was therefore driven largely by recovery of alveolar volume, which was 65% predicted at nadir. Membrane transfer per unit volume improved only later. This sequence is consistent with an acute phase in which consolidation reduced the ventilated parenchyma, followed by slower resolution of the alveolar-capillary component.

### 3.6. Histology and Its Timing

The cryobiopsy was obtained four months after presentation, after corticosteroid treatment and after radiological improvement, and is a point worth stating, while the patient was taking imatinib, having resumed it 27 days earlier. The fibrotic NSIP pattern therefore describes the residual and possibly still active phase rather than the acute injury, whose radiological signature at onset was consolidative and organizing-pneumonia-like. Because no tissue was obtained during the acute phase, we cannot establish that the initial process was histologically organizing pneumonia, nor that it evolved into the fibrotic NSIP seen four months later. What this case documents is an organizing-pneumonia-like radiological pattern followed by a fibrosing radiological pattern, with subsequent cryobiopsy demonstrating fibrotic NSIP; the connection between the two remains a hypothesis. Evolution from an organizing pattern towards fibrotic NSIP is recognized in drug-induced injury, where pulmonary fibrosis, non-specific interstitial pneumonia, hypersensitivity pneumonitis and organizing pneumonia are the patterns most commonly described [2,19]. A fibrosing NSIP pattern was likewise documented in nilotinib-associated ILD during surgical biopsy [20], and NSIP-like appearances have been reported in recent cases characterized by cryobiopsy [13,15]. For imatinib itself, the only previous NSIP description came from a surgical specimen and was cellular rather than fibrotic, with complete radiological resolution within a month of withdrawal [9]; the fibrotic variant seen here is consistent with the incomplete recovery observed in our patient and with the irreversible fibrosis reported by Zhang and colleagues after nine months of exposure [11].

### 3.7. A Mechanistic Paradox

Imatinib is a tyrosine kinase inhibitor with activity against platelet-derived growth factor receptors and has itself been used therapeutically for pulmonary fibrosis induced by other drugs, including bleomycin and gemcitabine [21,22]. That the same agent should cause an interstitial process may seem contradictory. Recent work on human precision-cut lung slices offers a plausible reconciliation: imatinib reduced α-smooth muscle actin and collagen expression in stimulated tissue yet showed significant toxicity towards unstimulated alveolar epithelial cells and lung fibroblasts, suggesting that direct epithelial exposure carries a non-negligible cost [23].

### 3.8. Implications for the Tyrosine Kinase Inhibitor Switch

ILD among Bcr-Abl TKIs has been reported predominantly with imatinib, which supports a compound-specific rather than a class effect and makes substitution a reasonable strategy [4]. Selection of a subsequent inhibitor after first-line failure follows established principles [24]. In this patient, the switch was driven by insufficient molecular response rather than by toxicity, and bosutinib was thus far tolerated without pulmonary recurrence. Two caveats apply: the observation period is short, and bosutinib itself is associated with pleural effusion and with pulmonary arterial hypertension [4]. Continued respiratory surveillance, including echocardiography, is therefore warranted rather than optional.

### 3.9. Study Limitations

The dates of imatinib administration were reconstructed from the patient’s account and could not be corroborated by dispensing records. Pulmonary function was measured in two laboratories using different reference equations and different methods for lung volumes, and values were not recalculated to Global Lung Function Initiative z-scores. Serum precipitins against avian antigens were not available, so the exclusion of hypersensitivity pneumonitis rests on exposure chronology. Only qualitative BCR-ABL testing at diagnosis was available to us; quantitative transcript values at the time of the switch were held by the referring hematology center, so the reported insufficiency of molecular response reflects the treating hematologist’s assessment rather than data verified by the authors. Kinase domain sequencing was not performed. The cryobiopsy yielded two small fragments from a single lobe, and sampling error could not be excluded. The temporal separation between drug withdrawal and corticosteroid initiation was two days, which precludes an unconfounded dechallenge. Finally, although serial pulmonary function testing documented the course here, a systematic review found that routine function testing performed poorly for the early detection of drug-induced lung disease, and it should not be relied upon as a screening strategy [25].

## 4. Conclusions

Imatinib-associated ILD may present with an organizing-pneumonia-like radiological pattern and subsequently evolve toward fibrosis, with later cryobiopsy showing a fibrotic NSIP pattern. Whether these findings represent different stages of a single pathological process could not be established histologically. Cryobiopsy can be performed safely when surgical biopsy is not appropriate. In this case, the causal association with imatinib was considered probable rather than proven, and hypersensitivity pneumonitis could not be definitively excluded. Where infection, drug and empirical treatment overlap, causality should be graded explicitly rather than asserted. Further studies are warranted to define the histopathological spectrum of tyrosine kinase inhibitor-associated interstitial lung disease and the safety of switching within the class.

## Figures and Tables

**Table 1:** Pulmonary function and gas exchange over time.

Variable	Jan 2026 (on Imatinib)	Apr 2026 (off Imatinib 30 d)	Jun 2026 (After Re-Administration)
FVC, L (% pred)	1.35 (49)	1.98 (82)	2.01 (73)
FEV_1_, L (% pred)	0.99 (46)	1.57 (79)	1.46 (68)
FEV_1_/FVC	0.74	0.79	0.76
TLC, L (% pred)	2.62 (55) ^a^	3.60 (75) ^b^	NR
RV/TLC, %	47.6	NR	NR
DLCO, % pred	30	NR	67
DLCO/VA, % pred	45	44	56
SpO_2_ at rest, %	91	NR	95
6MWD, m	NR	NR	330

^a^ Body plethysmography. ^b^ Multiple-breath nitrogen washout. NR, not reported. Measurements were obtained in two different laboratories using different reference equations; values are therefore expressed as percent predicted as reported locally and were not recalculated to Global Lung Function Initiative z-scores.

## Data Availability

The original contributions presented in this study are included in the article. Further inquiries can be directed to the corresponding author. The clinical data underlying this case report are not publicly available in order to protect patient privacy.

## References

[1] Boyle N., Butler M.W. (2026). Parsing the Patterns: Not All Drug-Induced Interstitial Lung Diseases Are Alike. Understanding Drug-Induced Interstitial Lung Disease in the Era of Targeted Cancer Therapy. Respirology.

[2] Freppel R., Benis A., Bonniaud P., Faillie J.L., Grandvuillemin A. (2026). Analysis of interstitial lung disease in pharmacovigilance databases: Coding challenges and interpretation biases-An update. Br. J. Clin. Pharmacol..

[3] Camus P., Fanton A., Bonniaud P., Camus C., Foucher P. (2004). Interstitial lung disease induced by drugs and radiation. Respiration.

[4] Weatherald J., Bondeelle L., Chaumais M.C., Guignabert C., Savale L., Jaïs X., Sitbon O., Rousselot P., Humbert M., Bergeron A. (2020). Pulmonary complications of Bcr-Abl tyrosine kinase inhibitors. Eur. Respir. J..

[5] Shah R.R. (2016). Tyrosine Kinase Inhibitor-Induced Interstitial Lung Disease: Clinical Features, Diagnostic Challenges, and Therapeutic Dilemmas. Drug Saf..

[6] Ohnishi K., Sakai F., Kudoh S., Ohno R. (2006). Twenty-seven cases of drug-induced interstitial lung disease associated with imatinib mesylate. Leukemia.

[7] Isshiki I., Yamaguchi K., Okamoto S. (2004). Interstitial pneumonitis during imatinib therapy. Br. J. Haematol..

[8] Carrillo-Esper R., Morales-Victorino N. (2009). Toxicidad pulmonar inducida por imatinib [Imatinib-induced pulmonary toxicity]. Gac. Med. Mex..

[9] Lee N.R., Jang J.W., Kim H.S., Yhim H.Y. (2015). Imatinib mesylate-induced interstitial lung disease in a patient with prior history of Mycobacterium tuberculosis infection. Korean J. Intern Med..

[10] Guzelkucuk Z., Isık M., Ozcan A.S., Demirkan T.H., Yaralı N. (2018). Imatinib-related interstitial lung disease. Br. J. Haematol..

[11] Zhang P., Huang J., Jin F., Pan J., Ouyang G. (2019). Imatinib-induced irreversible interstitial lung disease: A case report. Medicine.

[12] Troy L.K., Hetzel J. (2020). Lung cryobiopsy and interstitial lung disease: What is its role in the era of multidisciplinary meetings and antifibrotics?. Respirology.

[13] Morviducci M., Maraz F., Ravaglia C., Petrarulo S., Dubini A., Costantini M., Diano D., Blasi F., Palange P., Piciucchi S. (2025). Bispecific T-Cell Engagers-Induced Interstitial Lung Disease: Two Case Reports Confirmed by Transbronchial Lung Cryobiopsy. Respiration.

[14] Kaburaki S., Tanaka T., Murata A., Kamio K., Tanaka Y., Terasaki Y., Kasahara K., Seike M. (2024). The Histopathology of Abemaciclib-Induced Interstitial Lung Disease: A First Case Report with Transbronchial Lung Cryobiopsy. Breast Cancer Basic Clin. Res..

[15] Kaburaki S., Okada N., Tanaka T., Kamio K., Tanaka Y., Terasaki Y., Kasahara K., Seike M. (2024). Ixekizumab-induced interstitial lung disease: A case report confirmed by transbronchial lung cryobiopsy. J. Dermatol. Treat..

[16] Muto Y., Kuse N., Inomata M., Awano N., Tone M., Takada K., Fujimoto K., Bae Y., Kumasaka T., Izumo T. (2021). Drug-induced hypersensitivity syndrome caused by minodronic acid hydrate. BMC Pulm. Med..

[17] Bondue B., Pieters T., Alexander P., De Vuyst P., Patino M.R., Hoton D., Remmelink M., Leduc D. (2017). Role of Transbronchial Lung Cryobiopsies in Diffuse Parenchymal Lung Diseases: Interest of a Sequential Approach. Pulm. Med..

[18] Tamura M., Saraya T., Fujiwara M., Hiraoka S., Yokoyama T., Yano K., Ishii H., Furuse J., Goya T., Takizawa H. (2013). High-resolution computed tomography findings for patients with drug-induced pulmonary toxicity, with special reference to hypersensitivity pneumonitis-like patterns in gemcitabine-induced cases. Oncologist.

[19] Poschenrieder F., Stroszczynski C., Hamer O. (2014). Medikamenteninduzierte interstitielle Lungenerkrankungen. Radiologe.

[20] Cho J.Y., Lee O.J., Kwon J., Kim D., Shin Y.M. (2022). A case report of nilotinib-induced irreversible interstitial lung disease. Medicine.

[21] Fenocchio E., Depetris I., Campanella D., Garetto L., Schianca F.C., Galizia D., Grignani G., Aglietta M., Leone F. (2016). Successful treatment of gemcitabine-induced acute interstitial pneumonia with imatinib mesylate: A case report. BMC Cancer.

[22] Banakh I., Lam A., Tiruvoipati R., Carney I., Botha J. (2016). Imatinib for bleomycin induced pulmonary toxicity: A case report and evidence-base review. Clin. Case Rep..

[23] Bozzini S., Bozza E., Bagnera C., Morbini P., Lettieri S., Della Zoppa M., Melloni G., Saracino L., Belliato M., Meloni F. (2024). Assessment of Imatinib Anti-Remodeling Activity on a Human Precision Cut Lung Slices Model. Int. J. Mol. Sci..

[24] García-Gutiérrez V., Breccia M., Jabbour E., Mauro M., Cortes J.E. (2022). A clinician perspective on the treatment of chronic myeloid leukemia in the chronic phase. J. Hematol. Oncol..

[25] Bui A., Han S., Alexander M., Toner G., Irving L., Manser R. (2020). Pulmonary function testing for the early detection of drug-induced lung disease: A systematic review in adults treated with drugs associated with pulmonary toxicity. Intern Med. J..

